# Ca^2^^+^ release and buffering effects of synthetic hydroxyapatite following bacterial acid challenge

**DOI:** 10.1186/s12903-020-01080-z

**Published:** 2020-03-21

**Authors:** Fabian Cieplik, Christina M. Rupp, Stefanie Hirsch, Denise Muehler, Joachim Enax, Frederic Meyer, Karl-Anton Hiller, Wolfgang Buchalla

**Affiliations:** 1 Department of Conservative Dentistry and Periodontology, University Hospital Regensburg, Franz-Josef-Strauß-Allee 11, 93053 Regensburg, Germany; 2 Oral Care Research Department, Dr. Kurt Wolff GmbH & Co. KG, Bielefeld, Germany

**Keywords:** Biofilm, *Streptococcus mutans*, Synthetic hydroxyapatite, Oral care, Dental caries

## Abstract

**Background:**

Synthetic particulate hydroxyapatite (HAP; Ca_5_(PO_4_)_3_(OH)) is used as ingredient in oral care products but its effects on cariogenic biofilms are not clear yet. The primary mode of action of HAP may be acting as a calcium phosphate reservoir when deposited in oral biofilms and release Ca^2+^ and (hydrogen) phosphate ions upon bacterial acid challenge. The aim of this in vitro study was to test this hypothesis by investigating release of Ca^2+^ ions and potential buffering effects from HAP upon bacterial acid challenge in planktonic cultures and biofilms of *Streptococcus mutans*.

**Methods:**

Planktonic cultures of *S. mutans* were grown in BHI broth with 1% sucrose or with additional 5% HAP or 5% silica for up to 48 h. Separately, biofilms of *S. mutans* were grown in BHI for 72 h in total. After 24 h of this biofilm culture, either BHI alone or BHI with additional 0.5% HAP or 0.5% silica was added. After 48 h, BHI with 1% sucrose was added to allow bacterial acid formation. Ca^2+^ release was determined colorimetrically and pH measurements were performed using a pH electrode. For statistical analysis, non-parametrical procedures were applied (*n* ≥ 10; Mann-Whitney U test; α = 0.05).

**Results:**

Relevant release of Ca^2+^ was only evident in planktonic cultures or biofilms with HAP but not in both other groups (*p* ≤ 0.001). In suspended biofilms with HAP, median pH was 4.77 after 72 h and about 0.5 pH units higher as compared to both other groups (4.28 or 4.32, respectively; *p* ≤ 0.001).

**Conclusions:**

Under the tested conditions, synthetic HAP releases Ca^2+^ ions upon bacterial acid challenge and may also show some buffering capacity but further studies are needed to investigate whether the concentrations tested here can also be reached clinically in dental biofilms.

## Background

According to the most recent Global Burden of Disease study, published in November 2018, dental caries still represents the most prevalent disease worldwide affecting about 2.3 billion adults and 532 million children [1]. Its related treatment costs thus represent a major economic burden for public health care [2, 3].

Dental caries is a biofilm-mediated disease which leads to demineralization of dental hard tissues due to acid production from oral biofilm bacteria that metabolize fermentable carbohydrates to organic acids [4, 5]. This can lead to many different chemical microenvironments with localized pH gradients within a biofilm [6]. Consequently, carious lesions do not result from a simple and continuously aggravating cumulative mineral loss but rather from a very dynamic process comprising alternating periods of demineralization with net mineral loss and periods of remineralization with net mineral gain due to calcium (Ca^2+^) and (hydrogen) phosphate (mostly H_2_PO_4_^−^ and HPO_4_^2−^) ions becoming deposited into the pores of the carious lesion; therefore, progression or reversal of early carious lesions depends on whether the balance between demineralization and remineralization is shifted in one direction or the other [4, 5, 7].

Fluoride compounds are the keystone of all contemporary caries prevention concepts [4, 5, 7]. Accordingly, the topical use of fluoride in toothpaste at concentrations ≥1000 ppm F^−^ has been shown to have high-certainty evidence for its caries-protective effects over non-fluoridated placebo toothpaste [8]. However, populations with high caries risk, e.g. orthodontic patients, patients suffering from hyposalivation or older patients in general, may benefit from additional measures in order to enhance the caries-protective effects provided by fluoride [7, 9]. In this context, calcium-based strategies have been discussed as potential boosters for caries protection when combined with fluoride compounds [7, 9–11]. Despite the supersaturation of saliva with Ca^2+^ with respect to enamel at neutral pH [12], there is an undersaturation in plaque fluid compared to the dental hard tissues under acidic conditions that may be reduced by a controlled release of Ca^2+^ [9]. Furthermore, the bioavailability of Ca^2+^ and (hydrogen) phosphate ions often represents a limiting factor for remineralization upon topical fluoride application, particularly in patients suffering from hyposalivation [7, 13, 14]. Consequently, several calcium phosphates have been described as potential caries-preventive agents, e.g. hydroxyapatite (HAP), α−/*β*-tricalcium phosphate (α-TCP; *β*-TCP), and amorphous calcium phosphate (ACP) [7, 9, 10], which all differ with regard to their respective composition, crystallinity, molar Ca/P ratio, and solubility (for more details see ref. [15]). Among these calcium-based caries-preventive agents, casein phosphopeptide-amorphous calcium phosphate (CPP-ACP) has been studied intensively [7, 9, 10, 16], while recently several studies have been published investigating synthetic particulate HAP (Ca_5_(PO_4_)_3_(OH)) as another calcium phosphate-based compound [17–20]. HAP has often been proposed as a biomimetic agent for directly filling up micropores on demineralized tooth surfaces [17] and by reducing initial bacterial colonization on enamel surfaces [18]. However, in patients exhibiting high plaque scores the primary mode of action of HAP as an oral care agent may be to act as a calcium phosphate reservoir when deposited in oral biofilms, potentially releasing Ca^2+^ and (hydrogen) phosphate ions H_2_PO_4_^−^, HPO_4_^2−^ and PO_4_^3−^ upon bacterial acid challenge and thus maintaining a state of higher saturation with respect to these ions at the tooth surface [19, 20].

To test this hypothesis, this in vitro study served as a proof-of-principle investigation examining whether synthetic particulate HAP releases Ca^2+^ ions upon bacterial acid challenge when it is present at high concentrations in planktonic cultures or biofilms of *Streptococcus mutans*. Furthermore, potential buffering effects of HAP were investigated.

## Methods

### Test substances

Synthetic particulate hydroxyapatite (HAP; commercial grade; mean particle size: 4 μm) and particulate hydrated silica (SiO_2_·nH_2_O; commercial grade; mean particle size: 12 μm) were both provided by Dr. Kurt Wolff GmbH & Co. KG (Bielefeld, Germany). A recent scanning electron microscopic structural characterization of the HAP powder used here showed that the particles are micrometer-sized stable clusters of crystallites resembling the structure of human enamel [21]. Hydrated silica was used as control to exclude any impairments of particulate agents on bacterial growth or biofilm formation.

### Bacterial culture

*Streptococcus mutans* (DSM 20523; NCTC 10449) was obtained from the DSMZ (Deutsche Sammlung von Mikroorganismen und Zellkulturen, Braunschweig, Germany). Bacteria were grown and maintained on Schaedler Agar plates (provided by the Institute of Clinical Microbiology and Hygiene, University Hospital Regensburg, Germany) under aerobic conditions. Brain Heart Infusion (BHI) broth (Sigma-Aldrich, St. Louis, MO, USA) was used as basal nutrient broth. Planktonic cultures were prepared by selecting colonies, suspending them in 5 mL BHI and culturing over-night aerobically in order to obtain bacteria in the stationary phase of growth.

### pH and Ca^2+^ release in planktonic cultures

Over-night cultures of *S. mutans* were harvested by centrifugation (ROTINA 420 R, Hettich Lab Technology, Tuttlingen, Germany), washed in phosphate-buffered saline (PBS; Sigma-Aldrich, St. Louis, MO, USA) and resuspended in 1 mL BHI yielding an optical density (OD) of 1.0 measured at 600 nm by means of a spectrophotometer (Ultrospec 3300 pro, Amersham Biosciences, Amersham, UK). These suspensions were transferred to brand-new glass baffled flasks (Schikane-Kolben, Schott, Mainz, Germany) containing 20 mL BHI with 1% sucrose either alone or in combination with 5% (w/v) HAP or 5% (w/v) silica. The baffled flasks were incubated at 37 °C for a total culture period of 48 h on an orbital shaker (100 rpm), which kept HAP or silica particles dispersed during culture. At baseline, after 24 h and after 48 h, pH was measured by means of a pH meter (HI 2211, HANNA Instruments, Woonsocket, RI, USA) and release of Ca^2+^ was determined colorimetrically (Calcium assay kit ab102505; Abcam, Cambridge, UK) by measuring OD at 575 nm on a Varioskan® Flash microplate reader (Thermo Fisher Scientific, Waltham, MA, USA).

### pH and Ca^2+^ release in biofilms, CFU assay

Over-night cultures of *S. mutans* were centrifuged, resuspended in BHI yielding an OD of 0.1 measured at 600 nm and further diluted tenfold in BHI. Biofilms were formed in 24-well polystyrene culture plates (Corning® Costar®, Corning, NY, USA). Wells were filled with 1.5 mL BHI containing *S. mutans* and incubated at 37 °C under aerobic conditions. After 24 h, medium was carefully discarded and fresh BHI (untreated control) or fresh BHI with 0.5% (w/v) HAP or 0.5% (w/v) silica was added, and biofilms were incubated. After 48 h, medium was carefully removed and fresh BHI with addition of 1% sucrose was added to allow acid formation and biofilms were incubated for another 24 h.

After a total culture period of 72 h, the supernatants were carefully discarded and used for supernatant pH measurements and colorimetric determination of Ca^2+^ release (as described above). Then, biofilms were suspended in 1 mL 0.9% NaCl and transferred to Eppendorf tubes. These were placed in an ultrasonic water-bath chamber operating at 35 kHz (Sonorex Super RK 102 H, Bandelin, Berlin, Germany) for 10 min and vortexed (REAX top, Heidolph Instruments, Schwabach, Germany) for 5 s in order to separate aggregated bacteria. Then, these biofilm-derived suspensions were used for pH measurements and colorimetric determination of Ca^2+^ release (as described above). Tenfold serial dilutions (10^− 2^ to 10^− 7^) were prepared from the biofilms in 0.9% NaCl and aliquots (3 × 20 μL) were plated on Schaedler agar plates according to the method described by Miles et al. [22]. These were incubated anaerobically for 48 h, after which colony forming units (CFU) were evaluated.

### Data analysis

Results from pH measurements and colorimetric Ca^2+^ release assays are shown as medians, 1st and 3rd quartiles and were calculated using SPSS, v. 25 (SPSS Inc., Chicago, IL, USA) from the values of at least 10 (planktonic) or 12 (biofilm) independent duplicate experiments, respectively. For calculating Ca^2+^ concentrations from OD values, a standard was prepared according to the manufacturer’s guidelines (Calcium assay kit ab102505). OD values were fitted on a linear curve (r^2^ = 0.96) using Table Curve 2D (Systat Software Inc., San Jose, CA, USA). Data were analyzed statistically by applying non-parametric procedures (Mann-Whitney U test) for pairwise comparisons between groups on an α = 0.05 level of significance using SPSS. CFU results were calculated as medians, 1st and 3rd quartiles with SPSS from the values of at least 6 independent experiments, each performed in duplicate.

## Results

### pH and Ca^2+^ release in planktonic cultures

**Fig. 1:**
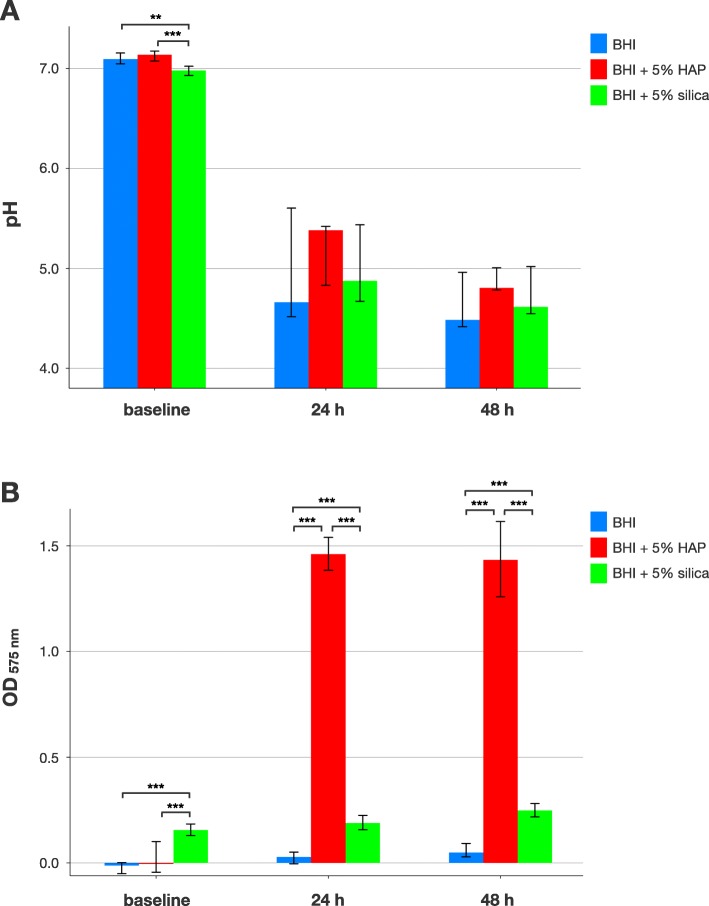
pH and Ca^2+^ release in planktonic cultures. Results from pH (**a**) and Ca^2+^ release measurements (**b**) in planktonic cultures of *S. mutans* at baseline and after 24 h and 48 h of culture. All results are depicted medians, 1st and 3rd quartiles from the values of at least 10 independent duplicate experiments. Asterisks indicate statistically significant differences between groups (** *p* ≤ 0.01; *** *p* ≤ 0.001)

Figure 1 shows pH and Ca^2+^ release in planktonic cultures of *S. mutans*. At baseline, median [1st; 3rd quartile] pH was 7.1 [7.05; 7.16] for BHI alone, 7.14 [7.08; 7.18] for BHI + 5% HAP and 6.98 [6.93; 7.02] for BHI + 5% silica, whereby the latter was found to be statistically significantly different compared to the other groups (*p* = 0.002). After 24 h of incubation, pH dropped to 4.66 [4.52; 5.6] for BHI alone, 5.38 [4.83; 5.42] for BHI + 5% HAP and 4.88 [4.67; 5.44] for BHI + 5% silica. After 48 h, pH further decreased to 4.49 [4.42; 4.96] for BHI alone, 4.81 [4.78; 5.01] for BHI + 5% HAP and 4.62 [4.55; 5.02] for BHI + 5% silica. There were no statistically significant differences at 24 h and 48 h of planktonic incubation between the groups (Fig. 1a).

Release of Ca^2+^ was evident for BHI + 5% HAP (median Ca^2+^ concentrations [1st; 3rd quartile] of 38.9 μg/mL [36.8; 41.0] at 24 h and 38.1 μg/mL [33.3; 43.1] at 48 h) but not for BHI alone (0.0 μg/mL [0.0; 0.06] at 24 h and 0.0 μg/mL [0.0; 1.1] at 48 h) and only very slightly for BHI + 5% silica (3.8 μg/mL [2.9; 4.8] at 24 h and 5.4 μg/mL [4.6; 6.3] at 48 h). Statistically significant differences were found between BHI + 5% silica and the other groups at all timepoints and between BHI + 5% HAP and the other groups at 24 h and 48 h (*p* ≤ 0.001 in all cases; Fig. 1b).

### pH and Ca^2+^ release in biofilms

**Fig. 2:**
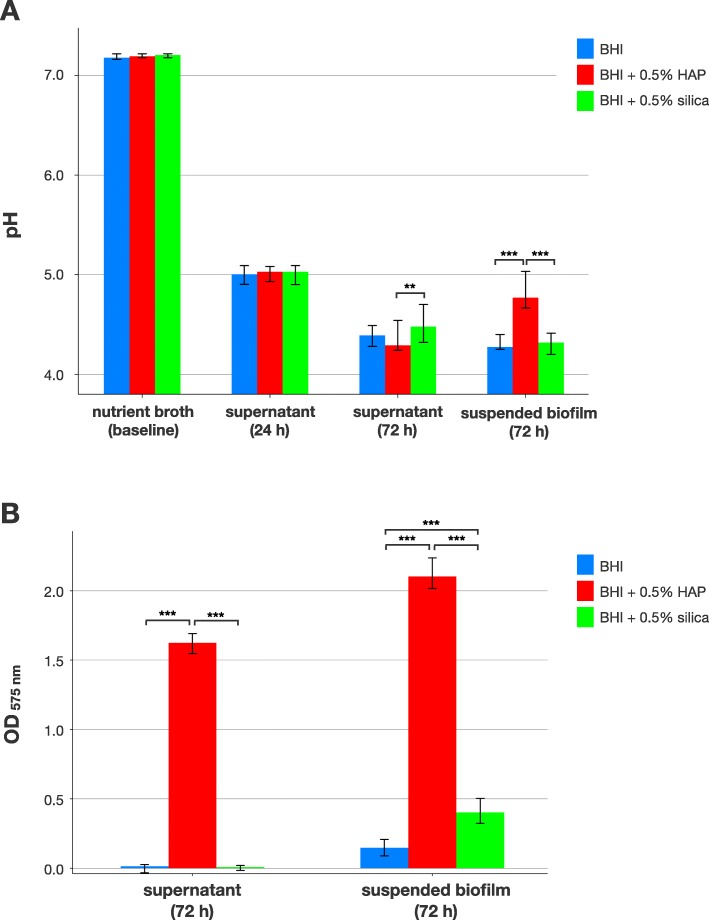
pH and Ca^2+^ release in biofilms. Results from pH (**a**) and Ca^2+^ release measurements (**b**) in *S. mutans* biofilms. pH was measured in the growth medium at baseline, in supernatants after 24 h of culture and in supernatants as well as suspended biofilms after 72 h of culture. Ca^2+^ release was measured in supernatants and suspended biofilms after 72 h. All results are depicted medians, 1st and 3rd quartiles from the values of at least 12 independent duplicate experiments. Asterisks indicate statistically significant differences between groups (** *p* ≤ 0.01; *** *p* ≤ 0.001)

Figure 2 shows pH and Ca^2+^ release in *S. mutans* biofilms. At baseline, median pH was between 7.18 and 7.21 for all groups. Supernatants collected after 24 h of biofilm culture exhibited median pH between 5.00 and 5.03 for all groups, which decreased to median [1st; 3rd quartile] pH 4.39 [4.28; 4.49] for BHI alone, 4.29 [4.24; 4.54] for BHI + 0.5% HAP and 4.48 [4.32; 4.7] for BHI + 0.5% silica for supernatants collected after 72 h of biofilm growth with a statistically significant difference between BHI + 0.5% HAP and BHI + 0.5% silica (*p* = 0.004). Suspended biofilms exhibited median pH 4.28 [4.25; 4.4] for BHI alone and 4.32 [4.2; 4.41] for BHI + 0.5% silica, while pH was statistically significantly higher for BHI + 0.5% HAP (4.77 [4.67; 5.03]); *p* ≤ 0.001; Fig. 2a).

Ca^2+^ release was detected in the BHI + 0.5% HAP group in supernatants collected after 72 h (median Ca^2+^ concentrations [1st; 3rd quartile] of 43.4 μg/mL [41.3; 45.3]) and in suspended biofilms (56.6 μg/mL [54.2; 60.3]), whereas no relevant Ca^2+^ release was found for BHI alone (0.0 μg/mL [0.0; 0.0] in supernatants and 2.6 μg/mL [1.0; 4.3] in suspended biofilms) or BHI + 0.5% silica (0.0 μg/mL [0.0; 0.0] in supernatants and 9.6 μg/mL [7.5; 12.4] in suspended biofilms. Differences were statistically significant between BHI + 0.5% HAP and the other groups in 72 h supernatants and between all groups in suspended biofilms (*p* ≤ 0.001 in all cases; Fig. 1b).

### CFU assay

All biofilms showed growth of viable bacteria exhibiting median [1st; 3rd quartile] CFU-numbers in the same order of magnitude with 1.4 × 10^7^ [8.8 × 10^6^; 2.1 × 10^7^] for BHI, 5.5 × 10^7^ [2.9 × 10^7^; 7.1 × 10^7^] for BHI + 0.5% HAP and 2.6 × 10^7^ [1.5 × 10^7^; 3.7 × 10^7^] for BHI + 0.5% silica CFU per biofilm.

## Discussion

The present in vitro study served as a proof-of-principle investigation for evaluating whether bacterial acid challenge can cause release of Ca^2+^ ions and consequent pH-buffering effects from the potentially caries-protective oral care agent HAP when it is present at relatively high concentrations in planktonic cultures and biofilms. For this purpose, planktonic cultures and biofilms of *S. mutans* served as model systems. *S. mutans* is well known for its role in the pathogenesis of dental caries and its ability to extensively produce extracellular polymeric substances that promote bacterial attachment and biofilm formation [23]. For this study, *S. mutans* was chosen rather than other caries-associated bacteria due to its pronounced acidogenic [24] and aciduric [25] properties.

In the first part of this study, planktonic cultures of *S. mutans* were grown either in pure nutrient broth or nutrient broth with 5% HAP or with 5% silica. The relatively high concentration of 5% was chosen analogously to a recent in situ study, which used a 5% HAP mouthwash and showed some slightly anti-adhesive effects of this mouthwash [18]. Silica is a frequently used toothpaste abrasive [26], and was included as control for excluding potential impairing effects of particles on bacterial growth or biofilm formation. Due to the experimental set-up (i.e. culture on an orbital shaker for keeping HAP or silica particles dispersed), it was inevitable to culture *S. mutans* under aerobic conditions. Ahn et al. described several biochemical and phenotypic changes in *S. mutans* during aerobic growth (including acidogenicity), but also concluded that *S. mutans* is highly adaptable to different environments [27]. On the contrary, Higuchi et al. showed that the growth of the *S. mutans* strain used in the present study (DSM 20523, NCTC 10449) was enhanced by oxygen and retarded by anaerobiosis. Furthermore, this specific strain also produced more lactate under aerobic conditions, whereas anaerobiosis led to heterolactic fermentation with production of mainly ethanol and formate [28]. Accordingly, pH values around pH 4.5 after 48 h clearly showed relevant acid production from *S. mutans* although these cultures may have mainly comprised bacterial cells in a stationary phase due to the rather high starting inoculum. Sucrose (at a concentration of 1%) was chosen as a carbon source in the nutrient broth because this has been found to be support the highest acid production from *S. mutans* [29, 30].

In the presence of HAP, there was a significant release of Ca^2+^ resulting in median Ca^2+^ concentrations in the nutrient broth of 38.9 or 38.1 μg/mL after 24 or 48 h respectively. Salivary Ca^2+^ concentrations have been reported to range between 20 and 55 μg/mL [11, 31–33]. This means that, under the given experimental conditions, Ca^2+^ release from HAP was in the same range as the concentration of human salivary Ca^2+^ and may thus be considered relevant, although HAP dispersed in planktonic bacteria does not directly resemble any natural situation in the oral cavity. On the other hand, the slight but significant Ca^2+^ increases found for the silica group as compared to the group with nutrient broth alone may be attributed to minor calcium impurities in the chosen silica. After 24 h and 48 h of planktonic culture, there was a tendency for slightly higher pH in the HAP group as compared to the other two groups.

These results could be affirmed in the biofilm experiments which may be more relevant with respect to the natural situation in vivo. Here, *S. mutans* biofilms were incubated for 72 h in total, whereby after 24 h either pure nutrient broth or nutrient broth with 0.5% HAP or with 0.5% silica were allowed to sediment on the preformed initial biofilms and after 48 h nutrient broth with 1% sucrose was added to promote bacterial acid production. The use of single-species *S. mutans* biofilms may be seen as a limitation here but polymicrobial biofilms may have caused problems in terms of species-species interactions that may have diminished acid production. Therefore, we chose a single-species biofilm model with an *S. mutans* strain that is known to produce high loads of lactic acid [28]. The concentration of 0.5% of HAP or silica, respectively, was chosen to mimic dilution effects as they may occur in the oral cavity after using a mouthwash with 5% HAP [18]. In contrast to previous works [34, 35], we used no filter-sterilized saliva here to promote biofilm formation as salivary Ca^2+^ ions (concentrations between 20 and 55 μg/mL [11, 31–33], as described above), may have impeded Ca^2+^ release measurements.

After 72 h of biofilm culture, there was a clear Ca^2+^ release corresponding to median Ca^2+^ concentrations of 43.4 or 56.5 μg/mL in the supernatants and suspended biofilms, respectively, while no such effects were found in either of the other groups. Therefore, HAP may act as a calcium reservoir when present in dental biofilms and release Ca^2+^ following bacterially induced pH drops, thus potentially reducing demineralization by providing supersaturation with respect to calcium phosphate in the plaque fluid. Zhang et al. investigated whether the presence of biofilms has effects on the treatment outcomes of nano-HAP and sodium fluoride [36]. They cultured biofilms on artificially demineralized enamel specimens and subjected them to a pH-cycling schedule with twice daily applications of nano-HAP or sodium fluoride. For nano-HAP treatment, they found an enhanced demineralization protection in specimens with biofilms during pH-cycling which they attributed to the high calcium content in the biofilms that may act as a diffusion barrier, potentially slowing the outflux of calcium and phosphate [36]. Accordingly, Shaw et al. found as early as 1983 that Ca^2+^ concentrations in plaque were statistically significantly higher in caries-free children (anterior plaque: 11.55 μg/mg dry weight; posterior plaque: 3.57 μg/mg) compared to children who had been highly caries-active within the last two years, exhibiting a mean DMFS of 25.9 (anterior plaque: 2.57 μg/mg; posterior plaque: 1.63 μg/mg) [31].

Interestingly, pH was found to be significantly higher (about 0.5 pH units) in suspended biofilms of the HAP group compared to the other two groups. The buffering effects observed for HAP may be explained by its chemical properties. Calcium phosphates in general are basic and release Ca^2+^ and (hydrogen)phosphate ions H_2_PO_4_^−^ and HPO_4_^2−^ under acidic conditions. The corresponding equation for HAP around pH 4.5 (like in the present study) is, as follows:

$$ {\mathrm{Ca}}_5{\left({\mathrm{PO}}_4\right)}_3\left(\mathrm{OH}\right)+7\ {\mathrm{H}}^{+}\rightarrow 5\ {\mathrm{Ca}}^{2+}+{\mathrm{H}}_2\mathrm{O}+3\ {\mathrm{H}}_2{{\mathrm{PO}}_4}^{-} $$

The release of H_2_PO_4_^−^ may act as an additional buffer system similar to the phosphate buffer system found in human saliva [5, 12]. An in vitro study showed that the buffering range of calcium phosphates is around pH 4 [37]. Thus, HAP may show its best buffering efficacy in highly acidic environments like cariogenic biofilms following carbohydrate ingestion as in the present study. Nedeljkovic et al. evaluated the buffering ability of dental restorative materials and included HAP discs as a control material [38]. They found the highest buffering capacity among the tested materials for HAP and observed higher pH increases for lower starting pH values, which they attributed to increasing dissolution of HAP with decreasing pH [38].

These findings are in accordance with Huang et al. who investigated the remineralizing effects of nano-HAP on demineralized bovine enamel under pH-cycling conditions [19]. They found that the surface microhardness increased and the integrated mineral loss significantly decreased as pH decreased, with the most effective group found at pH 4 [19]. In contrast, Zhang et al. found no increased remineralization in their study described above. They explained the discrepancy to the findings from Huang et al. by different components in their remineralization/demineralization buffers (mainly proteins) which may block the enamel surface to some extent [36]. In a recent in situ-study, Amaechi et al. found remineralization of artificially produced carious lesions on enamel blocks that were worn in situ on intra-oral appliances after use of a toothpaste containing 10% HAP [39].

Lately, it was also reported that application of CPP-ACP may have prebiotic-like effects on microbial ecology in highly cariogenic environments by increasing the buffering capacity of the biofilm and favoring growth of commensal species [40, 41]. Given the findings reported here, this may also hold true for other calcium phosphates like HAP.

Although these findings are promising, there are two limitations of this study that must be considered when interpreting its results: 1) HAP was sedimented onto the biofilms after 24 h of culture. So, it is not clear whether HAP accumulates in oral biofilms in vivo, although Vogel et al. already demonstrated the general possibility for accumulation of calcium phosphates in dental plaque when evaluating Ca^2+^ concentrations in plaque fluid after use of an α-TCP-containing chewing gum [11]. 2) HAP was added to the biofilms in a relatively high concentration (0.5%) that may exceed the concentrations found in dental biofilms in vivo. For these reasons, the present study only resembles “optimal conditions”. Future studies will have to show whether and to what extent synthetic HAP accumulates in oral biofilms following application of HAP-containing oral care products like mouthwashes. Furthermore, comparing Ca^2+^ release from different calcium phosphates like HAP, ACP or α−/*β*-TCP upon bacterial acid challenge may be an interesting topic for further studies.

## Conclusions

This in vitro study serves as a proof-of-principle investigation demonstrating that synthetic particulate HAP releases Ca^2+^ ions upon bacterially induced acid challenge when present in *S. mutans* biofilms at high concentrations. Furthermore, HAP showed a slight buffering capacity in biofilms of about 0.5 pH units. Future studies will have to show whether the HAP concentrations tested here can be reached clinically in dental biofilms upon application of HAP-containing oral care products.

## Data Availability

The datasets used and/or analyzed during the current study are available from the corresponding author on reasonable request.

## Abbreviations

HAP: Hydroxyapatite
α-TCP: α-tricalcium phosphate
*β*-TCP: *β*-tricalcium phosphate
ACP: Amorphous calcium phosphate
CPP-ACP: Casein phosphopeptide-amorphous calcium phosphate
BHI: Brain heart infusion
OD: Optical density
CFU: Colony forming units
